# Parental Attunement, Insightfulness, and Acceptance of Child Diagnosis in Parents of Children With Autism: Clinical Implications

**DOI:** 10.3389/fpsyg.2020.01849

**Published:** 2020-08-07

**Authors:** Magda Di Renzo, Viviana Guerriero, Giulio Cesare Zavattini, Massimiliano Petrillo, Lidia Racinaro, Federico Bianchi di Castelbianco

**Affiliations:** ^1^Institute of Orthophonology (IdO), Rome, Italy; ^2^Department of Dynamic and Clinical Psychology, Sapienza University of Rome, Rome, Italy

**Keywords:** parental attunement, insightfulness, acceptance of child diagnosis, autism spectrum disorders, parent–child interaction

## Abstract

Early parent–child relationships are an important factor influencing many domains of child development, even in the presence of autism. In this study, we investigated the associations between parent–child attunement during play, parental insightfulness, and parental acceptance of their child’s diagnosis of an autism spectrum disorder. A sample of 50 parents (26 mothers and 24 fathers) of 26 children aged between 24 and 58 months were videotaped during parent–child play interactions and then interviewed about what they thought had gone through their child’s head during the play interaction, and about their feelings and thoughts about their child’s diagnosis. Play interactions were evaluated using a coding protocol to assess parental attunement. The results showed that parents who were more able to accept their child’s diagnosis and to see things from their child’s perspective were more likely to also be attuned during play interactions with their children. These findings highlight the importance of studying the parental ability of insightfulness and acceptance of their child diagnosis of ASD for the implementation of intervention programs for supporting parental attunement and improving the interactions between the parents and the children with autism spectrum disorders.

## Introduction

Autism spectrum disorders (ASDs) are neurodevelopmental disorders characterized by deficits in social communication, and restrictive, repetitive behavioral patterns emerging early in child development. These children also show an intensified emotional reactivity and difficulties in emotion regulation (Samson et al., 2012).

A recent study found a relationship between children’s alexithymia and a reduction in parent–child interactions in the presence of a diagnosis of ASD, when compared to parents of typically developed children (Costa et al., 2019). Moreover, a 2019 review have revealed that parental verbal responsiveness to their children’s focus of attention predicted children’s expressive and receptive language (Edmunds et al., 2019). In this respect, considering that children with ASD may display poorer communicative behaviors than children with typical development, these fewer social interactions may lead to reduced learning opportunities with parents (Tager-Flusberg, 2016). Given the significance of difficulties in social relationships for these children, many researchers have argued for the need to better understand the role and quality of early relationships with primary caregivers (Crowell et al., 2019).

Research into parent–child dyads highlights the fact that social competence is an important factor in child development (Raver and Zigler, 1997; Vaughan Van Hecke et al., 2007; Denham et al., 2012; Domitrovich et al., 2017). Social competence is shaped within interactive, mutual exchanges as part of the development of early parent–child relationships (Feldman and Masalha, 2010), for which attuned parenting is fundamental (Landry et al., 2006; Leerkes et al., 2009). Parental attunement is a core dimension, defined as the parental ability to be responsive to child signals, understand them, and respond appropriately, while adjusting to the child’s needs (Stern et al., 1985; Stern, 1998; Schore, 2001; Zand et al., 2014). This competence emerges during parent–child interactions, laying the foundation for a shared and emotionally connoted experience, which represents a precursor for the development of the child’s mind, his/her abilities in self-regulation, and capacity to be engaged in relationships with others.

In research investigating the role of parental attunement in child development, little has involved samples of parents who have children with ASD. A study of 39 parents of children with different diagnoses (including autism) indicated that parents showing greater knowledge of child development were more likely to be attuned to their children. Greater parental attunement also predicted more positive attitudes toward child independence, which in turn predicted child social competencies (Zand et al., 2014). A pilot study examining parent–child physiological synchrony, that is the parent and child electrodermal activity measured during naturalistic free play, highlighted that higher ASD symptoms were associated with lower levels of parental emotional attunement and synchrony (Baker et al., 2015). In a sample of 40 preschoolers with ASD and 40 matched typically developing (TD) peers, children’s ability to self-regulate and mother and father parental disciplinary style were explored (Ostfeld-Etzion et al., 2016). The study confirmed what was already emerging in the relevant literature (Feldman and Klein, 2003; Hirschler-Guttenberg et al., 2015), namely, that parents of children with ASDs used the same parental disciplinary style of parents of TD children, and that a more supportive parental disciplinary style was associated with more child self-regulated compliance. According to a 2017 study in an ASD group, mother–child dyadic interactions were more engaged in mismatched emotion-engagement states and children spent more time exclusively with objects than the dyads in the TD group (Guo et al., 2017). Another recent study used a narrative methodology to study fathers’ stories of play interaction with their children with ASD aged between 5 and 12 years old. Three narratives emerged from the fathers’ stories (action, adjustment, and acceptance), and among them, acceptance narratives were more likely in fathers showing resistance to societal norms of play, acceptance of, and attunement to their children’s play interests (Mitchell and Lashewicz, 2018).

An important contribution to understanding the roles played by the parents of children with ASD has been made by Oppenheim and Koren-Karie (Oppenheim et al., 2001; Koren-Karie and Oppenheim, 2018) with the introduction of the concept of parental insightfulness. This refers to the parental ability to see things from the child’s point of view. Previous studies have shown that insightful mothers were more sensitive within their interactions with their children, and these mothers are also more likely to have children with secure attachments (Koren-Karie et al., 2002). Furthermore, insightful mothers were found to display higher levels of positive parenting during interactions with their children, regardless of the number of stressful life events experienced by the mother (Martinez-Torteya et al., 2018) when compared to non-insightful mothers. Higher levels of cooperation and co-parenting in triadic interactions when both parents were insightful were also identified, and no differences were found between mothers and fathers in their ability to see things from the child’s point of view (Marcu et al., 2016). A recent study (Feniger-Schaal et al., 2019) on 38 mothers of children with intellectual disabilities found that 41% of the mothers showed positive insightfulness and that better capacity for insightfulness was associated with better maternal sensitivity^1^ behavior during mother–child interactions when compared to non−insightful mothers.

When comparing a group of clinically depressed vs. non-depressed mothers, Ramsauer et al. (2014) showed lower sensitivity and insightfulness toward their child, in depressed mothers. Based on clinical theorizing, in the presence of a mental illness, parental ability to display attunement/sensitivity and insightfulness toward a child may be somewhat impaired, which may negatively influence parent–child relationship (Oppenheim and Koren-Karie, 2009; Carter and DelCarmen-Wiggins, 2020). However, to our knowledge, there are no studies investigating these variables in a sample of children with ASD.

Within the population of children with ASD, a 2008 study showed that maternal insightfulness did not depend on the severity of ASD or the level of child functioning. Overall, 42% of mothers were found to be insightful and 58% were found to be non-insightful, regardless of the severity of ASD (Oppenheim et al., 2008). Furthermore, in a sample of 39 children with ASD and their mothers, maternal insightfulness and child secure attachment at preschool age predicted better adaptation to developmental tasks, such as school, 4 and 8 years later (Dolev et al., 2014). Consistent with this, a recent systematic review on autism and attachment showed that maternal sensitivity and insightfulness support the development of secure attachment in children with ASD (Kahane and El-Tahir, 2015).

As stated by Oppenheim et al. (2009), in cases of the diagnosis of severe pathology, the study of the parental state of mind should include not only insightfulness but also the acceptance or resolution of the diagnosis as “seeing things from the child’s point of view must also include understanding and accepting the challenges associated with the child’s diagnosis” (Oppenheim et al., 2009, p. 519). Resolution is the process of the integration of this information/emotion [about their child diagnosis] within the parents’ representational systems of themselves as parents, of their child, and of the relationship with their child (Pianta and Marvin, 1993, p. 3). Receiving a diagnosis may cause disruption or damage to normal maternal fantasies about a child (Pouillaude, 2018) and negatively interfere with a parent’s acceptance of the child’s diagnosis and their investment in the child–parent relationship. Lack of acceptance can interfere with the parental ability to integrate the representations of a “healthy” and “ill” child, and with the possibility of focusing their attention on the present and their relationship with the actual child (Marvin and Pianta, 1996; Pianta et al., 1999; Zavattini, 2016).

In studies with children with different diagnoses, the proportion of parents with acceptance of the child’s diagnosis varies from 36 to 81% (Lord et al., 2008; Milshtein et al., 2010; Barak-Levy and Atzaba-Poria, 2013; Yirmiya et al., 2015; Dolev et al., 2016; Baiocco et al., 2017). Parental acceptance of the child’s diagnosis does not seem to depend on the time passed since receiving the diagnosis (Pianta et al., 1996; Lord et al., 2008; Hutman et al., 2009; Oppenheim et al., 2009; Milshtein et al., 2010; Kearney et al., 2011; Lecciso et al., 2013; Popp et al., 2014), the child’s gender (Marvin and Pianta, 1996; Schuengel et al., 2009; Kearney et al., 2011; Yirmiya et al., 2015; Krstić et al., 2016), or parental gender (Lord et al., 2008; Schuengel et al., 2009; Milshtein et al., 2010; Barak-Levy and Atzaba-Poria, 2013; Yirmiya et al., 2015). Instead, it has been found that maternal acceptance of the child’s diagnosis relates to more sensitive caregiving during social play (Dolev et al., 2016) and a better maternal perception of their physical health (Reed and Osborne, 2019). Failure in accepting the child diagnosis is linked to higher maternal distress (Lord et al., 2008; Kearney et al., 2011; Krstić et al., 2015), parental depression (Kearney et al., 2011; Krstić et al., 2015), lower levels of emotional support (Sheeran et al., 1997), greater use of avoidance strategies (Freda et al., 2016), and lower maternal sensitivity (Dolev et al., 2016). Few studies have investigated both paternal and maternal acceptance of child diagnosis, but those have found significant gender differences. Mothers low in acceptance, but not fathers, reported more parental negative feelings and more negative impacts of the child’s disease on their social life and marriage (Milshtein et al., 2010). Fathers reported higher levels of couple satisfaction if mothers were able to accept their child diagnosis (Sheeran et al., 1997) and mothers were more prone to use an emotional coping style while fathers tended to use a cognitive coping style when they talked about the experience of receiving the diagnosis of the child’s illness (Barak-Levy and Atzaba-Poria, 2013).

As for research on the parents of children with ASD, Milshtein et al. (2010) studied 60 fathers and 61 mothers and found that almost 43% were classified as acceptance of the child diagnosis and that for mothers, the acceptance of the diagnosis was associated with a better perception of the child and the impact of raising a child with a disability on family life. Another study (Lecciso et al., 2013) with a sample of 21 mother–child dyads with high-functioning autism showed that accepting mothers of their child diagnosis were better able to see themselves and their children as mental agents, to think of themselves as a secure base, and to not avoid the negative aspects of the relationship. The maternal ability to accept the child diagnosis was associated with the type of diagnosis: in contrast to the results by Milshtein et al. (2010), the researchers found that mothers of children with high-functioning autism were more likely to be accepting of their child diagnosis than mothers of children with Asperger’s syndrome.

Seventy-seven parents of recently diagnosed children with ASD were the participants of a study (Poslawsky et al., 2014) that found that parental acceptance of the child diagnosis (also known as Resolution) was associated with less severe autistic symptoms, and demonstrated a substantial stability of the resolution classification relating to the child’s diagnosis after 7 months from the first evaluation. Yirmiya et al. (2015) also examined the stability of resolution classification over time (3 years after the first evaluation) among 78 mothers and fathers of children with ASD. At time 2 (3 years after the first evaluation), mothers’ acceptance of the child diagnosis was significantly predicted by an increase in maternal anxiety, an increase in the children severity of symptoms, and a longer duration of time since they received the diagnosis. A 2016 paper presented data from a sample of 46 mothers of children with ASD aged between 2 and 8 years, demonstrating that accepting mothers were more likely to be sensitive to their children during play and reported less psychological parental distress and fewer child symptoms compared to mothers low in acceptance (Dolev et al., 2016). A recent study on 84 mothers of children newly diagnosed with ASD showed that mothers low in acceptance had a worsening of maternal health status (in terms of their perception of their symptoms) after 1 year from the time of their child diagnosis, and they perceived to have a poorer health status when compared to mothers more able to accept their child diagnosis (Reed and Osborne, 2019).

Finally, some studies have investigated both parental insightfulness and the acceptance of child diagnosis. A 2009 study of 67 mothers and their children with ASD did not identify a significant association between these two variables, highlighting instead that insightful mothers were more synchronous than non-insightful mothers during play, while mothers able or not able to accept their child’s diagnosis did not significantly differ from each other in synchronous behavior during play (Hutman et al., 2009). The maternal ability to accept child diagnosis and maternal insightfulness were both associated with a secure attachment classification in children with ASD (Oppenheim et al., 2009). A further paper also demonstrated that maternal sensitivity mediated the association between insightfulness/maternal acceptance of the diagnosis and child attachment in a sample of 45 preschool children with ASD (Oppenheim et al., 2012).

The studies discussed above show that, to our knowledge, only one study investigated the relationships between attunement, insightfulness, and acceptance of the child diagnosis, in the presence of a diagnosis of autism for children, focusing exclusively on mothers. The aim of the present study was therefore to examine the relationships between these three aspects of parental functioning, on both mothers and fathers. We hypothesized that the parents of children with ASD who are insightful and able to accept their child diagnosis are more likely to be attuned with their children during play interaction than parents low in their ability to accept their child diagnosis and insightfulness.

## Materials and Methods

### Sample

Participants in this study were 50 parents (24 fathers and 26 mothers) of 26 children who had been diagnosed within the past 3 months of study participation with ASD or being at risk for autism due to a diagnosis of global developmental delay. Children ranged in age from 24 to 58 months (*M* = 34.36, *SD* = 8.65) and the total sample comprised 23 (88%) males and 3 (12%) females. Autistic risk was calculated for children under 30 months using the Toddler Module of the Autism Diagnostic Observation Schedule-2 (ADOS-2; Lord et al., 2012a, b). The children with a diagnosis of ASD were 8 (Module 1 Pre-verbal of ADOS-2) and the children with a diagnosis of Global Developmental Delay (GDD) with a risk for autism were 18 (Toddler Module of ADOS-2). Before participating in this study, parents and children have received from 0 to 3 months of intervention. Only one parent refused to participate in the study. The average age of mothers was 38.20 years (SD = 5.51) and the average age of the fathers was 41.38 years (*SD* = 9.09). Of the parents, 80% were Italian and the remaining 20% were from other countries. Concerning educational level, 14.3% of parents obtained a middle school diploma or lower grade, 46.9% a high school diploma, and 38.8% a university degree or higher (Table 1).

**TABLE 1:** Descriptive statistics.

	*N* (%)		*M*	DS
	Females	Males		
Parents’ gender	26 (52%)	24 (48%)		
Children’s gender	3 (12%)	23 (88%)		
Parents’ age			39.755 (Years)	7.570
Children’s age			34.360 (Months)	8.651
Length of treatment			1.040 (Months)	1,228
**Educational level^#^**				
Middle school diploma or lower	7 (14.3%)			
High school diploma	23 (46.9%)			
University degree or higher	19 (38.8%)			
**Severity of the symptoms**				
Mild	5 (19.2%)			
Moderate	10 (38.5%)			
Severe	11 (42.3%)			
**Acceptance of child diagnosis**				
Resolved	24 (48%)			
Unresolved	26 (52%)			
**Insightfulness**				
Insightful	27 (54%)			
Non-insightful	23 (46%)			
**Attunement**				
Attuned	26 (52%)			
Unattuned	24 (48%)			
**Acceptance of diagnosis/Insightfulness**				
(A) Resolved/Insightful	21 (42%)			
(B) Unresolved/Non-insightful	20 (40%)			
(C) Unresolved/Insightful or Resolved/Non-insightful	9 (18%)			

*^#^One missing data.*

### Procedure

The parents were recruited at the Institute of -*Blinded for Peer Review*- between 2017 and 2018. Parent–child dyads were videotaped during play interactions lasting 15 min. The play interactions used for coding the DAOS were the same for assessing parental AI. Parents were then asked to complete a questionnaire and to respond to a videotaped interview lasting about 30–45 min. The clinicians who communicated the diagnosis to the families were different from the team of psychologists in the present study. One of the authors of this study is the clinician who administered the ADOS-2, during the assessment for the diagnosis of autism. No children had received a diagnosis before the assessment at our center. The “at-risk group” was made only by toddlers under 30 months of age and that is why there was no diagnosis of ASD.

Parents were recruited after they have received a diagnosis of ASD or Global Developmental Delay (GDD) with a risk for autism for their children. The child diagnosis was communicated to parents after the diagnostic process carried out at the Institute of Orthophonology (IdO) of Rome (Di Renzo et al., 2015). The Reaction to Diagnosis Interview was administered with regard to the actual diagnosis they had (ASD or GDD with a risk for autism). At the moment of participating in this study, parents and children have received from 0 to 3 months of intervention at our clinical institute. The intervention consisted of 10 h of treatment per week including 6 h of child individual/group therapy, 2 h of school observation and counseling, and 2 h of parental psychological support, carried out by different clinicians than those who conducted the present study (Di Renzo et al., 2020b).

This study was not submitted to an Ethical Committee for ethical review and approval because it is suggested but not mandatory in our legislation. In accordance with articles 5, 7, and 9 of the Italian Ethical Code for Psychologist, a written informed consent to participate in this study was provided by the participants’ legal guardian of children. Before participating in the study, parents were asked to sign an informed consent indicating the methods, possible risks, and purpose of the study, as well as being given the possibility of refusing to participate further at any time, in accordance with the Helsinki Declaration (World Medical Association, 2013).

### Instruments

The *Autism Diagnostic Observation Schedule*, Second Edition (ADOS-2; Lord et al., 2012a, b; Colombi et al., 2013) is a semi-structured, standardized assessment of communication, social interaction, play, and restricted and repetitive behaviors for children aged between 12 months to adulthood. It presents various activities that elicit behaviors directly related to a diagnosis of ASD. By observing and coding these behaviors, we obtained information relating to two areas: Social Affect (AS) and Restricted and Repetitive Behaviors (RRBs). Critical behaviors in the area of Social Affect, quantified in the coding algorithm, receives a score ranging from 0 to 2, where 0 indicates normotypic behavior, 1 indicates a behavior that is present but atypical and/or not very flexible, and 2 indicates an anomaly or an absence of such behaviors. The RRBs follow a progressive numerical coding based on their frequency and intensity increasing from 0 to 2. The overall score is given by summing AS and RRBs. The ADOS-2 includes five modules: the Toddler Module, for children between 12 and 30 months of age who do not have language or who do not consistently use phrase speech; Module 1, for children from 31 months and older who do not consistently use phrase speech; Module 2: for children of any age who use phrase speech but are not verbally fluent; Module 3, for verbally fluent children and young adolescents; Module 4, for verbally fluent older adolescents and adults. The ADOS-2 has good psychometric properties confirming its usefulness in distinguishing individuals with ASD from other clinical groups (Mazefsky and Oswald, 2006; Gotham et al., 2007, 2009; Lord et al., 2012a, b; Hus and Lord, 2014; Esler et al., 2015).

The *Reaction to Diagnosis Interview* (RDI) (Pianta and Marvin, 1993) is a brief, 15 min interview, aimed at examining parental resolution of the loss/trauma associated with the experience of receiving a child diagnosis of disability or chronic illness. The RDI assesses this acceptance (or lack of acceptance) through videotaping and then coding an individual parent’s responses to six standardized questions with specific probes investigating beliefs, memories, and emotional reactions of parents to the news of the child’s illness and any changes that have occurred over time. The coding yields the major classifications of Resolved or Unresolved, plus several sub-classifications within each major classification (Pianta and Marvin, 1993). The Resolved parents are those accepting the diagnosis of their child and can describe with balance the changes that may have occurred following the communication of the diagnosis, without continuing to look into the past or to question the possible causes of what happened (Marvin and Pianta, 1996). They show greater acceptance of the situation over time and can describe the difficulties of the disease and the specific characteristics of their child. Unresolved parents provide inconsistent descriptions of the diagnosis experience. They may produce distorted stories, which highlights an inability to describe the reality of the situation, or the story appears confused and it is difficult for the encoder to follow the thread of the discourse. Parents can also experience difficulty in managing their feelings related to the memory of the diagnosis experience and show themselves to be emotionally overwhelmed by anger or pain, or depressed and/or lost in their memories.

The *Insightful Assessment* (IA) (Koren-Karie and Oppenheim, 2004) is a video replay procedure for assessing parental insightfulness. The procedure involves an initial phase in which the parent and the child are videotaped during three different moments of interaction. Then, the parent is invited to watch brief video clips and interviewed regarding his/her child’s thoughts and feelings. The evaluation allows each parent to be assigned one of the following categories: Positively Insightful (PI) in which the parent shows that he can describe the child in a complex way and can focus on his internal world; One-sided (Os) in which the parent has a one-dimensional view – positive or negative – of the child and the relationship; Disengaged (De) in which the parent shows a lack of emotional involvement in the description of the child and the relationship; and Mixed (Mx) in which no single, coherent parent strategy emerges (Koren-Karie and Oppenheim, 2018). In our study, parents were divided according to whether they fell into the broad Insightful and Non-insightful classifications (which includes the Os, De, and Mx classifications).

The *Dyadic Attunement Observation Schedule* (DAOS; *under validation*) is an observational measure of parent–child interaction during play. The DAOS observation schedule was used for scoring parent–child dyads videotaped during play interactions. Parents were invited to play with their children as if they were at home. The clinician gave the parents the instructions of creating three different circumstances lasting about 5–10 min each: a time of free play and two structured playtimes (i.e., blocks and sponge ball). This observational assessment consists of eight scales: 1. joint attention, 2. body, 3. interaction, 4. space sharing, 5. play sharing, 6. authonomy, 7. emotional regulation, 8. understanding child mental states. Each scale has a score ranging from 0 to 3 and the final coding allows parents to be assigned one of two categories: Attuned or Unattuned. Attuned parents can adapt their bodies to respond to their child’s signals with combined and alternating use of their space (remaining close to/far, next to/face to face). They are generally able to play with their child by activating a body dialog made up of gestures, sounds, and eye gazing, supporting an interactive exchange in which they organize role switching and involving the child with sufficient participation (without intrusiveness). Attuned parents are also able to facilitate their child’s actions without overlap with the child, with the aim of increasing his autonomy and supporting his skills so that the child may experience new actions. They can offer their emotional availability to the child by co-regulating emotions when the child is not able to regulate these by himself. They are also able to recognize and repair moments of failed attunement. Unattuned parents, on the other hand, do not play with their children by activating body dialog (they sometimes look like clumsy or inhibited), and they are not very proactive in involving their child in play. They show little or no shared and alternative use of space, remaining close to/far, next to/face to face), tending to overpower the child or to withdraw following demands for play. These parents may show a strongly passive role, feeling inadequate, and unable to contain and regulate their child’s emotions during difficult periods in the interaction. They are powerless to repair moments of failed attunement.

At present, the DAOS has currently been used with children with typical development, learning disabilities, speech disorders, anxiety disorders, and emotion regulation problems. The measure is under validation.

### Data Analysis

We used chi-squared tests to examine differences in parental attunement, acceptance of diagnosis, insightfulness, parental gender, parental educational level, and child severity of symptoms. The variable “severity of symptoms” ranging from 1 to 5 (1 = no evidence, 2 = minimum, 3 = mild, 4 = moderate, 5 = severe symptomatology), was created on the basis of the scores from ADOS-2 and clinical observations of the deficits in the quality of communication and relational behaviors calibrated upon children’s age.

We used *t*-tests to determine any significant differences between Attuned/Unattuned, Resolved/Unresolved, and Insightful/Non-insightful parents with respect to the variables “children age” and “length of the treatment.” In order to investigate our hypothesis that both Resolved and Insightful parents were more attuned with their children with ASD during play interactions, we created a combined variable Resolution/Insightfulness, similar to the approach used by Oppenheim et al. (2009). Three groups of (A) Resolved/Insightful (21), (B) Unresolved/Non-Insightful (20), and (C) Unresolved/Insightful or Resolved/Non-Insightful (9) parents were formed.

A 3 × 2 cross-tabulation was performed to examine differences between this new variable and parental attunement. We used the likelihood ratio (LR) when our data did not meet the assumption of having at least 80% of the cells with an expected count of over 5 for the chi-squared tests.

The differences between the two groups of children with ASD and autistic risk were calculated for the study variables, showing no statistically significant differences between the two groups (χ^2^ = 1.923, *p* = 0.166 for attunement; χ^2^ = 1.087, *p* = 0.297 for insightfulness; χ^2^ = 0.855, *p* = 0.355 for the reaction to diagnosis). We therefore considered the entire sample in further analyses without distinguishing between the two groups.

In line with Rosner (2010), reported by Dogan and Dogan (2015), ICC < 0.4 indicates poor dyadic relationship, so we assumed our dyads had poor relationships for the three main variables of our study (acceptance of diagnosis, insightfulness, and attunement), and we considered mothers and fathers separately for statistical analysis.

## Results

### Descriptive Analysis

As shown in Table 1, 26 parents were classified as Unresolved (15 fathers and 11 mothers); 23 parents were Non-Insightful (16 fathers and 7 mothers), and 24 parents were Unattuned (13 fathers and 11 mothers). Children were assigned to a group according to the severity of symptoms as follows: 3 = mild (19.2%), 4 = moderate (38.5%), and 5 = severe (42.3%). No children were assigned to the groups 1 = no evidence or 2 = minimum.

We examined the associations between insightfulness, acceptance of the diagnosis, and parental attunement with the study variables: parental gender, severity of the child’s symptoms, and level of parental education. No differences emerged between mothers and fathers for parental acceptance of the diagnosis (*p* = 0.153) or parental attunement (*p* = 0.402). Significant differences emerged between mothers and fathers relating to insightfulness (*p* = 0.005), with mothers being more insightful than fathers. No significant association was found between the severity of the child’s symptoms and RDI classification *p* = 0.055), parental insightfulness (*p* = 0.869), or parental attunement (*p* = 0.942). No significant association emerged between parental educational level and RDI (*p* = 0.051), or parental attunement (*p* = 0.145). The association between parental educational level and parental insightfulness was statistically significant (*p* = 0.006), with insightful parents more likely to have a university degree or higher and non-insightfulness parents more likely to have a high school diploma (Table 2).

**TABLE 2:** Descriptive statistics, associations, and group differences with acceptance of the child diagnosis, insightfulness, and attunement.

	Acceptance of child diagnosis (RDI)		χ^2^	Insightfulness (IA)		χ^2^	Attunement (DAOS)		χ^2^
						
	Resolved (% of the total)	Unresolved (% of the total)		Insightful (% of the total)	Non-insightful (% of the total)		Attuned (% of the total)	Unattuned (% of the total)	
Mothers	15 (30%)	11 (22%)	2.039	19 (38%)	7 (14%)	7.936*	15 (30%)	11 (22%)	0.703
Fathers	9 (18%)	15 (30%)		8 (16%)	16 (32%)		11 (22%)	13 (26%)	

			**LR**			**LR**			**LR**

Middle school diploma or lower^#^	2 (4%)	5 (10%)	5.950	4 (8%)	3 (6%)	10.360*	3 (6%)	4(8%)	3.859
High school diploma	8 (16%)	15 (31%)		7 (14%)	16 (33%)		9 (18%)	14 (29%)	
University degree or higher	13 (27%)	6 (12%)		15 (31%)	4 (8%)		13 (27%)	6 (12%)	

			**LR**			**LR**			**LR**

Mild	8 (16%)	2 (4%)	5.803	6 (12%)	4 (8%)	0.283	5 (10%)	5 (10%)	0.120
Moderate	7 (14%)	13 (26%)		10 (20%)	10 (20%)		11 (22%)	9 (18%)	
Severe	9 (18%)	11 (22%)		11(22%)	9 (18%)		10 (20%)	10 (20%)	

	**M (DS)**	**M (DS)**	***t***	**M (DS)**	**M (DS)**	***t***	**M (DS)**	**M (DS)**	***t***

Children’s age	33.250 (7.320)	35.385 (9.753)	0.869	33.518 (7.029)	35.348 (10.316)	0.742	34.808 (7.408)	33.875 (9.966)	0.378
Length of treatment	1.083 (1.380)	1.308 (1.436)	0.678	1.259 (1.534)	1.130 (1.254)	0.247	1.269 (1.538)	1.125 (1.262)	0.219

*p < 0.01. ^#^One missing data. LR, Likelihood Ratio.

Furthermore, no significant differences emerged between Resolved/Unresolved (*p* = 0.389), Insightful/Non-insightful (*p* = 0.462), and Attuned/Unattuned (*p* = 0.707) parents according to child age. No significant differences emerged between Resolved/Unresolved (*p* = 0.501), Insightful/Non-Insightful (*p* = 0.806), and Attuned/Unattuned (*p* = 0.828) parents according to the length of the treatment (Table 2).

### Associations Between Parental Acceptance of the Child Diagnosis/Insightfulness and Attunement

We checked the associations between the variable Resolution/Insightfulness and the study variables that were significantly associated with Insightfulness: level of education and gender of the parents. The association with the educational level was statistically significant (LR = 10.269, *df* = 4, *p* < 0.05) with Resolved/Insightful parents more likely to have a university degree or higher, and Unresolved/Non-insightful parents more likely to have a high school diploma. The association with the parental gender was not statistically significant (LR = 5.844; *df* = 2; *p* = 0.054) for parental gender.

A 3 × 2 Resolution/Insightful × Attuned crosstab (Table 3) showed a significant association between the variables (LR = 10.157, *df* = 2, *p* < 0.01). Parents classified as both Insightful and Resolved were more likely to be Attuned during the play interaction with their children than parents in the other two groups, and parents classified as both Non-Insightful and Unresolved were more likely to be Unattuned during the play interaction with their children than parents in the other two groups.

**TABLE 3:** Parental acceptance/insightfulness categories and attunement as percentages of the total sample.

	Attuned (% of the total)	Unattuned (% of the total)	LR
(A) Resolved/Insightful	15 (30%)	6 (12%)	
(B) Unresolved/Non-insightful	5 (10%)	15 (30%)	10.157*
(C) Unresolved/Insightful or Resolved/Non-Insightful	6 (12%)	3 (6%)	

*p < 0.01.

## Discussion

These findings support our hypothesis that parents high in acceptance of their child diagnosis and insightful are more likely to be attuned to children with ASD during play interactions than those low in acceptance and insightful. Parental abilities include understanding their child’s point of view and accepting the experience of having received a child’s diagnosis of ASD. In addition, being able to focus attention on the present and their relationship with the child, together with the ability to understand the child’s perspective taking into consideration his/her mental states, wishes, and difficulties, all appear to be associated with being responsive to a child’s signals and responding to these while appropriately adjusting for their needs.

From our clinical experience, we can assume that the parents able to accept their child diagnosis may better contrast the desires associated to the fantasies about his or her child as “healthy” (Pouillaude, 2018), protecting the child from the projection of unreal desires associated with him/her, or the parent manages to overcome the image of himself as the “parent of an autistic child” and that of the child as an “autistic child,” allowing both of them to access a process of individuation and psychic growth. Non-insightful parents are likely to have a rigid and unidimensional (positive or negative) perception of their child’s behavior and motivations, may show a lack of emotional involvement or interest providing only short and limited descriptions of the child, or may be very hostile, angry, and concerned about the child (Koren-Karie and Oppenheim, 2018).

Our data lay in the findings from the studies that, within the attachment framework, have shown that in mother–child dyads with the presence of a diagnosis of ASD, the ability to accept and elaborate the experiences of the diagnosis together with the capacity of insightfulness was associated with a secure attachment in children (Oppenheim et al., 2009). Furthermore, the relationship between insightfulness, acceptance of the diagnosis, and child’s attachment was mediated by maternal sensitivity (Kahane and El-Tahir, 2015). The acceptance of the child diagnosis along with insightfulness may favor parental ability to be responsive to child signals because they allow the parents to establish a relationship with the “real child,” that is, the one whom the parent meets and experiences in terms of strengths and weaknesses and the potential for his/her development. In cases of a severe diagnosis of the child, the parents may become frustrated and disappointed once confronted the “real child” with the “imaginary child,” that is the one dreamed of during pregnancy.

Thus, attunement ability can be proposed to promote a secure attachment allowing “the infant to perceive a sense of being accepted and recognized, which facilitates social adjustment and a positive psychological functioning” (Manini et al., 2013). Furthermore, as suggested in the *Introduction*, children’s social competence may be positively influenced by attuned parenting, supporting an aspect usually inadequate in children with ASD. This hypothesis should be verified in future research through longitudinal studies. Another interesting finding is that parents low in acceptance or in insightfulness are more likely to be attuned during play interactions with their children, suggesting a possible protective factor of at least one of the two parental abilities to understand child’s point of view or to accept the child diagnosis.

The percentage of parents who have accepted the child diagnosis experience and those demonstrating parental insightfulness are consistent with what emerges in other studies with parents of children with ASD (Hutman et al., 2009; Oppenheim et al., 2009; Milshtein et al., 2010; Lecciso et al., 2013; Yirmiya et al., 2015; Dolev et al., 2016). However, some differences to the published findings also emerged in the current study. In our study, the parental acceptance of child diagnosis was not associated with parental gender, child age, or parental educational level. No significant differences emerged for the severity of the children symptoms, in contrast to previous studies that found that a worsening of the ASD levels of functioning, along with other variables, predicted maternal acceptance of the child diagnosis (Yirmiya et al., 2015) and that the severity of the ASD diagnosis was associated with parental acceptance of the diagnosis (Poslawsky et al., 2014). A possible explanation for these differences concerns the selection of measures that are used to identify the level of severity, such as questionnaires, interviews, or observational tools. The use of different tools can make it difficult to compare the results obtained in different studies. However, we assume that parental acceptance of the diagnosis is associated with parental resilience and previous emotional stability rather than the severity of the child’s symptoms, allowing parents to find creative solutions even in the face of serious clinical scenarios.

We found that maternal insightfulness did not depend on the severity of symptoms or child age as ascertained in other studies (Oppenheim et al., 2008). However, our data showed a statistically significant association between parental educational level and parental insightfulness in the direction of higher educational level for insightful parents. This is consistent with findings from the study by Oppenheim et al. (2009) in a sample of mothers of children with ASD, indicating that mothers classified as insightful had a higher level of education than mothers classified as non-insightful. However, this pattern has not been confirmed in samples of mothers of children with typical development patterns (Oppenheim et al., 2001; Koren-Karie et al., 2002), suggesting that this relationship may be specific to samples of children with ASD (Oppenheim et al., 2009). We assume that a higher level of education could function as a protective factor in understanding the child’s internal world when it seems that the child deviates from typical functioning, or that a broad cultural background could help parents adapt their resources to the needs of the child. Moreover, a statistically significant association emerged between educational level and the combined variable Resolution/Insightful, with parents both accepting the child diagnosis and insightful more likely to have a university degree or higher, and parents both less able to accept the child diagnosis and less insightful more likely to have a high school diploma.

Finally, significant differences emerged between the insightfulness of mothers and fathers, suggesting that mothers are more insightful. This finding is in contrast with that from another study that tested whether mothers and fathers differ in insightful ability using a low-risk sample of parents of young children with typical development (Marcu et al., 2016). This probably highlights a specific aspect of our sample that should be explored further in future work. It is possible that some fathers may experience greater difficulties than mothers since, in general, they spend less time caring for children (Dyer et al., 2009; Hartley et al., 2014). Some research indicates that many fathers want to increase their levels of involvement in child care if supported on this path (Rankin et al., 2019), which may lead to them feeling frustrated if having less chance of developing an understanding of their child.

This study has several methodological strengths given that narratological and observational measures are less vulnerable to the willingness of participants to provide information or to provide a personal view of the information collected as compared to questionnaires. A further strength is that the literature has often failed to consider the role of fathers whereas we directly tested this. Nonetheless, this study has also some limitations that should be taken into account as they could reduce the generalization of the results. These include the small number of parents who participated, the specific diagnosis of the children involved (risk for autism relating to global developmental delay and autism spectrum disorder), and the use of one measure that is not yet validated in the literature (the Dyadic Parent–Child Attunement Observation Schedule). Literature provides several observational instruments to measure parental attunement, especially within attachment theory researchers. As mentioned above, the debate on attunement is still open and the authors vary in their formulation of this construct (Mesman and Emmen, 2013). The tool we used for the assessment of the parental attunement, which is currently being validated, was specifically built for assessing interaction within parent and child with autism, guided by our theoretical basis and specific therapeutic intervention, focused on children body and sensory processing to promote the ability to be responsive to others’ signals, understanding them and replying to them appropriately (Di Renzo, 2017). To overcome these limitations, future studies should involve a larger and less heterogeneous sample and include additional measurements of parental attunement. Furthermore, given the lack of information on the child’s level of development and the physical and mental health of the parents in this study, future research should investigate the relationship between these variables and the acceptance of the child’s diagnosis, insightfulness, and attunement. Finally, we want to report the cross-sectional design, the use of categorial rather than continuous variables, and the use of parents of the same children as further limitations of our study.

## Conclusion

The results presented in this study provide some insights into potential clinical work with the mothers and fathers of children with ASD. Studying the parental ability of insightfulness and acceptance of a child diagnosis of ASD has enriched our understanding of the processes underlying the interactions of these parents with their children. These aspects should be addressed through intervention programs for parents. At the Institute of Orthophonology (IdO) support for parents has been incorporated into the D.E.R.B.B.I. intervention (known in full as the Developmental, Emotional Regulation and Body-Based Intervention) within the Turtle Project (Di Renzo et al., 2016). The project combines various interventions offered to children and parents including child assessment (Di Renzo et al., 2019), counseling for parents, clinical sessions with the professionals who work with the child, thematic seminars and experiential workshops, mothers/fathers–child in care settings, and groups of parents (Di Renzo et al., 2020a).

The importance of starting and supporting a process of acceptance relating to the child diagnosis (Guerriero et al., 2017a, b; Guerriero and Di Folco, 2017; Freda et al., 2019; Waizbard-Bartov et al., 2019) and parental insightfulness could support the relational experiences that determine the child “way of being” that is strongly connected to the non-verbal aspects of parental communication, especially parental attunement (Di Renzo, 2017). According to Trevarthen and Delafield-Butt (2013), responsive and attuned communication and a pattern of timed and sensitive actions can compensate for children experiencing repetition of uncertain and anxious attempts, when psychomotor attunement with perceptive and motor experiences become confused (LaGasse and Hardy, 2013). The basis of this hypothesis is the importance of considering the close interaction between dyadic function and specific parenting abilities in the formation of the psychic structure and the self-regulating abilities of the child (Beebe et al., 1999).

The results of this study also help us to better understand some of the discrepancies between mothers and fathers, which could give useful indications in planning group interventions for parents of different genders.

To date, only a few studies have investigated the needs of parents of children with ASD while paying particular attention to fathers, their involvement in child therapy, and direct involvement in an intervention (Hartley and Schultz, 2015; Rankin et al., 2019). In the present study, we documented that, in our sample at least, mothers are more insightful than fathers, making it understandable that when children show behaviors that are difficult to manage and understand, as in the case of children with ASD, paternal insight may be inadequate. This aspect should, therefore, be considered as the main goal of group therapy aimed at fathers, while monitoring over time the usefulness of such an approach in supporting fathers’ ability to “see things from their child’s point of view” (Koren-Karie and Oppenheim, 2018, p. 223).

## Data Availability Statement

The raw data supporting the conclusions of this article will be made available by the authors, without undue reservation, to any qualified researcher.

## Ethics Statement

Ethical review and approval was not required for the study on human participants in accordance with the local legislation and institutional requirements. Written informed consent to participate in this study was provided by the participants’ legal guardian/next of kin.

## Author Contributions

MD and VG conceived the study concept. GZ and VG designed the model and developed the theory. MP and LR recruited the sample and administered the assessments. VG coded the interviews and analyzed the data. FB helped in supervise the project and with MD they provided critical revisions of the findings. All authors discussed the results and contributed to the final manuscript.

## Conflict of Interest

The authors declare that the research was conducted in the absence of any commercial or financial relationships that could be construed as a potential conflict of interest.

## Footnotes

1 In this context with sensitivity, we mean “the caregiver’s ability to understand and recognize child’s signals. In particular, this term refers not only to the parental ability to interpret the emotional and physical states expressed from the early hours of life, but also to the willingness to provide a sufficiently adequate response in terms of timing and contents” (Di Folco et al., 2016, p. 72). We are aware that the debate on attunement is still open. Specifically, the terms *attunement* and *sensitivity*, which have been carefully described within different theoretical paradigms, not without facing some confusion, were proposed with different conceptual terms (Mesman and Emmen, 2013; Di Folco et al., 2016). Maternal sensitivity appeared initially thanks to the observations conducted by Ainsworth (1967) and is generally used within attachment researchers to describe an ability that emerges within the parent–child relationship. However, the term *attunement* was first described by the psychiatrist and psychoanalyst Daniel Stern (Stern et al., 1985) and refers to individual’s ability to share affect, empathize, and appropriately respond to another person, not necessarily the child.
