# Corrigendum: Parental Attunement, Insightfulness, and Acceptance of Child Diagnosis in Parents of Children With Autism: Clinical Implications

**DOI:** 10.3389/fpsyg.2020.593327

**Published:** 2020-09-15

**Authors:** Magda Di Renzo, Viviana Guerriero, Giulio Cesare Zavattini, Massimiliano Petrillo, Lidia Racinaro, Federico Bianchi di Castelbianco

**Affiliations:** ^1^Institute of Orthophonology (IdO), Rome, Italy; ^2^Department of Dynamic and Clinical Psychology, Sapienza University of Rome, Rome, Italy

**Keywords:** parental attunement, insightfulness, acceptance of child diagnosis, autism spectrum disorders, parent–child interaction

In the original article, there was an error. The acronym “**D.E.R.B.B.I. intervention” was expanded to “Development Emotional Relation Body-Based Intervention**”.

An error was also made, referring to “the Institute of -Blinded for Peer Review-”

A correction has been made to ***Conclusion, Paragraph Number 1***:

The results presented in this study provide some insights into potential clinical work with the mothers and fathers of children with ASD. Studying the parental ability of insightfulness and acceptance of a child diagnosis of ASD has enriched our understanding of the processes underlying the interactions of these parents with their children. These aspects should be addressed through intervention programs for parents. At the Institute of Orthophonology (IdO) support for parents has been incorporated into the D.E.R.B.B.I. intervention (known in full as the Developmental, Emotional Regulation and Body-Based Intervention) within the Turtle Project (Di Renzo et al., 2016). The project combines various interventions offered to children and parents including child assessment (Di Renzo et al., 2019), counseling for parents, clinical sessions with the professionals who work with the child, thematic seminars and experiential workshops, mothers/fathers–child in care settings, and groups of parents (Di Renzo et al., 2020a).

The authors apologize for this error and state that this does not change the scientific conclusions of the article in any way. The original article has been updated.
